# Cancer-Immunity Cycle and Therapeutic Interventions- Opportunities for Including Pet Dogs With Cancer

**DOI:** 10.3389/fonc.2021.773420

**Published:** 2021-11-19

**Authors:** Samantha K. Von Rueden, Timothy M. Fan

**Affiliations:** ^1^ Department of Veterinary Clinical Medicine, University of Illinois at Urbana-Champaign, Champaign, IL, United States; ^2^ Cancer Center at Illinois, University of Illinois at Urbana-Champaign, Urbana, IL, United States

**Keywords:** comparative oncology, pet dogs and cats, cancer immunotherapy, spontaneous model, immune activation

## Abstract

The tumor-immune interplay represents a dynamic series of events executed by cellular and soluble participants that either promote or inhibit successful tumor formation and growth. Throughout a tumor’s development and progression, the host organism’s immune system reacts by generating anti-cancer defenses through various incremental and combinatorial mechanisms, and this reactive orchestration is termed the cancer-immunity cycle. Success or failure of the cancer-immunity cycle dictates the fate of both host and tumor as winner or loser. Insights into how the tumor and host immune system continuously adapt to each other throughout the lifecycle of the tumor is necessary to rationally develop new effective immunotherapies. Additionally, the evolving nature of the cancer-immunity cycle necessitates therapeutic agility, requiring real-time serial assessment of immunobiologic markers that permits tailoring of therapies to the everchanging tumor immune microenvironment. In order to accelerate advances in the field of immuno-oncology, this review summarizes the steps comprising the cancer-immunity cycle, and underscores key breakpoints in the cycle that either favor cancer regression or progression, as well as shaping of the tumor microenvironment and associated immune phenotypes. Furthermore, specific large animal models of spontaneous cancers that are deemed immunogenic will be reviewed and proposed as unique resources for validating investigational immunotherapeutic protocols that are informed by the cancer-immunity cycle. Collectively, this review will provide a progressive look into the dynamic interplay between tumor and host immune responses and raise awareness for how large animal models can be included for developing combinatorial and sequenced immunotherapies to maximizing favorable treatment outcomes.

## Introduction

Immuno-Oncology (IO) is a ballooning therapeutic landscape that holds great promise for improving long-term outcomes in both human and canine cancer patients. Currently, IO has been heralded as a tremendous advancement for treating cancer patients with particular immunogenic histologies, whereby exploiting the host immune system’s inherent antineoplastic machinery can be harnessed sufficiently to eliminate cancer cells entirely. However, the full benefit of immunotherapy remains unrealized, as only a minority of treated patients achieve lasting remission durations (1). While profoundly life-changing for some, there is urgent need to expand the benefits of IO across a larger swathe of cancer patients. Intuitively, maximal benefit of IO approaches will require the development of innovative combinatorial strategies (2, 3), as well as the preclinical evaluation in suitable and immunocompetent model systems (4–6).

In order to rationally design immunotherapeutic interventions, it is necessary to understand the dynamic interplay that occurs between host immunocytes, tumor cells, and the associated tumor microenvironment (7). As such, the optimal tailoring of immunomodulatory strategies, both temporally and spatially, requires comprehension of the cancer-immunity cycle which predicates how anticancer immune responses are generated and propagated (8). When functioning properly, the cancer-immunity cycle is a series of steps that are conducive to successful priming, activation, infiltration, and tumor specific targeting of cytotoxic T lymphocytes to mount a tumor inhibiting immune response (8–10). However, in cases of tumor growth, one or more steps in the cancer-immunity cycle succumb to tumor suppressive mechanisms, and thus, fail to provide protective anti-cancer immunity. Given the complex and dynamic interplay that evolves throughout the cancer-immunity cycle, faithful recapitulation of this intricate immunobiologic process requires the inclusion of sophisticated tumor models.

In addition to highly sophisticated rodent and 3D modeling approaches for evaluating novel immunotherapeutic strategies, pet dogs present a complementary, yet unique opportunity to aid in the advancements of IO because dogs provide an immunologically outbred, spontaneously developing, heterogenous tumor model that in many respects parallels humans (11–14). Importantly, naturally-occurring tumors in pet dogs possess remarkable similarities with regards to biologic behavior, histologic features, genetic mutations, and response to therapy, as compared to specific cancers in people (11–14). Given the spontaneous course of cancer development under operative immune mechanisms, pet dogs treated with interventional strategies at different steps of the cancer-immunity cycle have the potential to serve as valuable model systems for realizing the science and best clinical practices necessary in generating robust anticancer immune responses sufficient for improving the management of primary and metastatic tumor lesions (13, 15).

## Cancer-Immunity Cycle

The cancer-immunity cycle describes the generation of anticancer immune reactivity as a cyclic process operating in a self-propagating manner (8, 16). Mechanistically, the cancer-immunity cycle can be divided into sequential critical steps required for effective immunity against cancer cells beginning with *Step 1*- release of cancer cell antigens; *Step 2*- cancer antigen presentation; *Step 3*- priming and activation in secondary lymphoid organs; *Step 4*- trafficking of activated T cells to tumors; *Step 5*- infiltration of T cells into tumors; *Step 6*- recognition of cancer cells by tumor-infiltrating T cells; and *Step 7*- T cell-mediated selective targeting and killing of cancer cells. As a consequence of cancer cell killing by activated T cells, additional tumor antigens are released into the tumor microenvironment (TME), which then further amplifies subsequent rounds of the cancer-immunity cycle in a feed-forward manner.

The cancer-immunity cycle can be initiated through the production of tumor neoantigens as a consequence of tumorigenesis (17–20). High mutational burden is a signature attributed to many inflamed tumor phenotypes given that as genomic instability amplifies across time, so does the chance for generating non-synonymous point mutations that code for novel immunogens (21–24). However, favorable outcomes of *Step 1*, will not be achieved in the absence of other immune stimulating factors such as pro-inflammatory cytokines and immunogenic molecules released from dying cancer cells. *Step 2* involves tumor specific antigen presentation *via* MHC class I and MHC class II molecules on specialized antigen presenting cells, namely dendritic cells. Following activation, dendritic cells will migrate away from the local TME, and *Step 3* occurs in tumor draining lymph nodes, where a high volume of T cells can become exposed to tumor specific- or associated- antigens presented by dendritic cells and become primed and activated against tumor cells. Following priming and activation, *Step 4* involves the trafficking of activated tumor specific T cells back to the TME. In *Step 5*, diapedesis opens the flood gates to infiltrating tumor-specific T cells into the TME, so that primed effector T cells can recognize and bind to tumor antigens presented on MHC class I molecules in *Step 6*. Together, this culminates to *Step 7*, in which cytotoxic T cells kill tumor cells *via* perforin and granzyme release or through FasL : Fas ligation. Importantly, by incorporating serial biomarker assessments that accurately reflect the immune status of the tumor and microenvironment, it becomes possible to identify any defective step(s) in the cancer-immunity cycle and provides opportunity to restore normal functionality through directed immunotherapeutic interventions.

## Immune Profile Phenotypes

**Figure 1:**
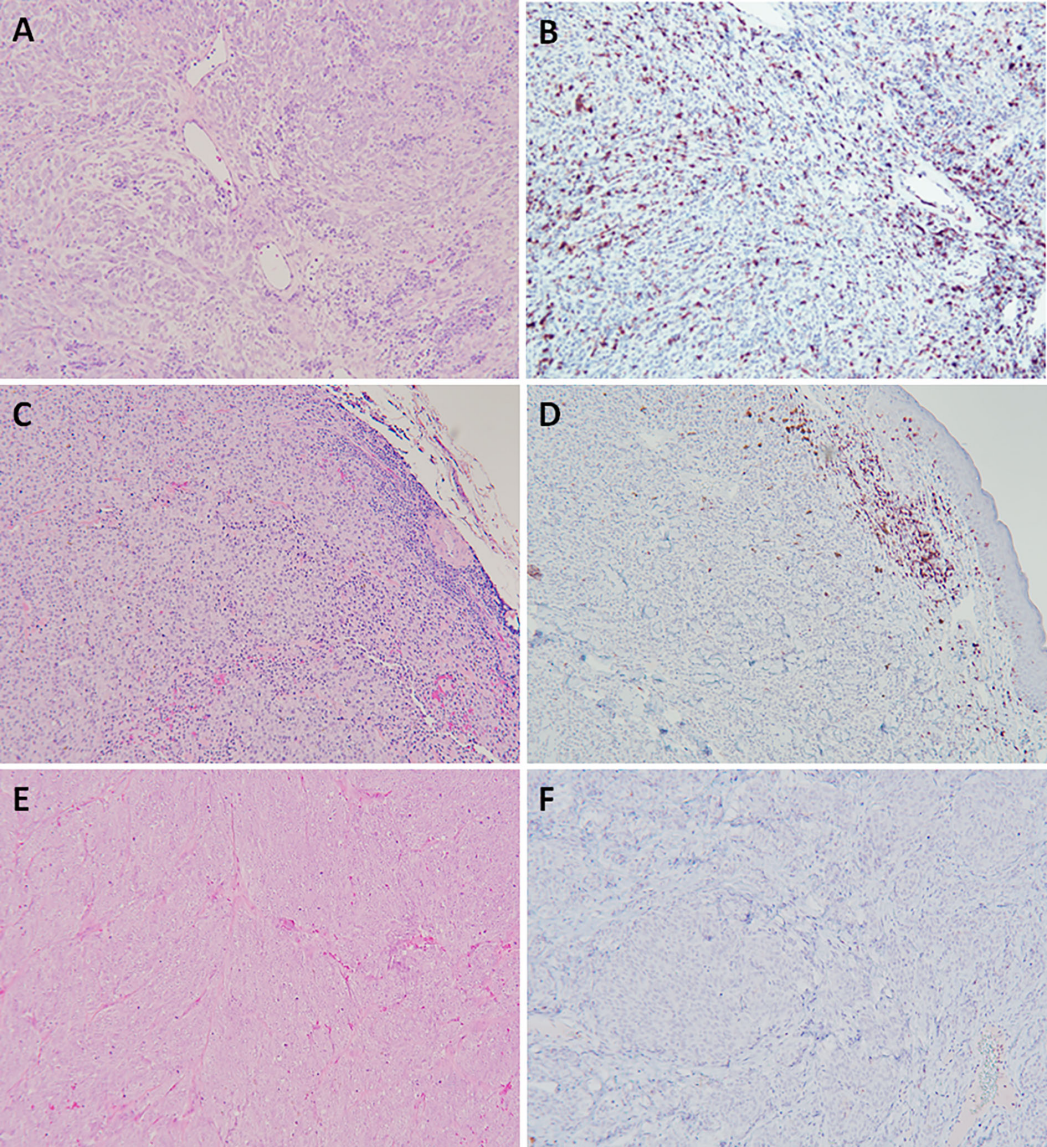
Naturally arising canine oral malignant melanoma with differences in immune profiles hallmarked by vastly differing quantity and spatial distribution of cellular infiltrates (H&E), and specifically CD3^+^ T lymphocytes (IHC, nova red chromogen). Representations of immune-inflamed **(A, B)**, immune-excluded **(C, D)**, and immune-desert **(E, F)**. Magnification 100x.

Given the multiple coordinated steps required for optimal cancer-immunity cycling, it is expected that tumor cells and associated TME can dramatically shape the localized immune milieu, including the quantity, quality, and composition of tumor-associated immunocytes, spatial and temporal kinetics of immune infiltration, and the functionality and competency of effector cells. Collectively, these cellular and morphologic hallmarks can be categorized into distinct profiles termed immune-desert, immune-excluded, and immune-inflamed phenotypes (**Figure 1**) (25). These immune profiles of tumorous lesions can guide the rational institution of immunomodulatory strategies for rectifying key breaks in the cancer-immunity cycle with consequent induction of robust anticancer immune responses. The clinical relevancy and predictive value of immune phenotype profiling has been underscored for the last decade, where positive responses to immunotherapeutic manipulations have been partially hinged upon the establishment and/or existence of an immune-inflamed TME.

Expanding the concept of “cold” versus “hot” tumors and classification into immune-desert, immune-excluded, and immune-inflamed, these phenotypes are defined by immune infiltration status reflecting the extent and topography predominantly of T lymphocytes within the TME, among other immune cells (26, 27). These three phenotypes span across an immune spectrum, with immune-desert representing a lower “cold” immunodeficient boundary, the immune-inflamed defining an upper “hot” immunoreactive boundary, and immune-excluded falling in between these 2 immune landscape extremes. The immune-desert is considered a non-inflamed tumor that has succumb to extreme tumor immunosuppressive mechanisms (28–30), and characterized by the seldom presence of tumor infiltrating lymphocytes (TILs) within tumor parenchyma or stroma. There is minimal to complete absence of CD8^+^ T cells, scant expression of PD-L1, low mutational burden, and only slight expression levels of MHC molecules (31). Collectively, the immune-desert phenotype represents a non-reactive TME that is permissive for tumor progression and poorly responsive to immunotherapeutic interventions (31–33). The immune-excluded phenotype can also be classified as a non-inflamed tumor, albeit, with some indication that a previous immune response was generated, yet ineffective largely due to physical microanatomic barriers created by the invasive margins delineating the tumor (31, 34–36). In the immune-excluded phenotype, rather than penetrating the cancerous parenchyma, T cells are confined within the dense stromal network surrounding the immediate tumor mass. The microanatomic constraints of the immune-excluded phenotype thwart successful immune surveillance and effector T cell attack of tumor cells. Lastly, the immune-inflamed profile is characterized by a significant and successful infiltration of T lymphocytes into the tumor parenchyma. Distinctively, both CD4^+^ and CD8^+^ T lymphocytes can be detected in close proximity to tumor cells, as well as the loco-regional liberation of pro-inflammatory cytokines that promote the activation and migration of tumor antigen specific T cells (36, 37). The immune-inflamed phenotype has increased mutational burden bearing a larger range of cancer derived antigens and tumor-specific neoantigens that effector T cells can recognize as non-self and selectively target (34, 37). Counterintuitively, tumors categorized as “hot” with an immune-inflamed phenotype can also express the highest levels of the immune inhibiting molecule, PD-L1. However, upregulation of PD-L1 is a compensatory immunomodulatory setpoint mediated by IFN-γ secreted from activated CD8^+^ and CD4^+^ T cells, which counteracts rampant immune reactivity through the production of PD-L1 and indoleamone-2,3-dioxygenase (IDO) immunosuppressive proteins (34). Moreover, IFN-γ is a major inducer of the chemokines, CXCL9 and CXCL10, which function as trafficking directors for NK cells and activated T cells to the tumor (38). Therefore, while the presence of CD8^+^ T lymphocytes remains integral to tumor regression, continuous exposure to tumor associated antigens induces an intrinsic regulatory feedback loop and ultimately leads to T cell exhaustion (9, 34, 39, 40).

Along with PD-L1, other checkpoint molecules including lymphocyte activation gene 3 (LAG3) and T cell immunoglobulin mucin (TIM3) are expressed on T cells in response to IFN-γ resulting in T cell suppression. As such, multi-pronged blocking strategies for LAG3, TIM3, and/or PD-L1 might synergistically amplify effector T cell responses by inactivating T cell suppression and promoting T cell activation (41–43). Although T cells are an important feature distinguishing the three immune phenotypes from one another, other immune cells also participate in the TME. Trafficking of NK cells and dendritic cells alongside T cells promotes inflamed tumor microenvironments, and their existence has been associated with improved outcomes in certain solid tumors, such as neuroblastoma (44). Likewise, invariant Natural Killer T (iNKT) cells enhance anti-tumor immune surveillance by recognizing specific lipid antigens, and once bound to their cognate ligands, iNKT cells engage both the innate and adaptive immune systems that establish broad anti-tumor immunity and contributes to an inflamed phenotype. The potential clinical importance of iNKT cells is highlighted by their attenuated presence in cancer patients suffering from disease progression in various tumor histologies including multiple myeloma, prostate cancer, and a spectrum of other solid tumors (45).

Correlating immune profiles with the tumor-immunity cycle, it is reasonable to postulate that immunotherapeutic manipulations that restore activation of immune exhausted T cells have potential to reinvigorate effective immune attack against tumors harboring an immune-inflamed profile; and tumor immune escape and immune profiles favoring either immune-desert or immune-excluded result from dysregulation of the tumor-immunity cycle and consequent impeded function of tumor specific T cells. Whether immunocyte dysfunctions are mediated through impaired migration, exhaustion or failure to become activated, T cells remain central and indispensable for protective tumor immune surveillance. By understanding the molecular mechanisms that contribute towards a tumor’s conversion into a T cell immune-inflamed phenotype or other non-inflamed phenotype (desert or excluded), the rational institution of immunotherapies which target these mechanisms can be employed to foster an activated T cell response for improved anticancer activities (9, 10, 37, 39).

### Factors Influencing Tumor Immune Profiles

Many immunotherapies namely checkpoint blockade, yield successful results with durable responses. However, positive responses are achieved in only a subgroup of cancer patients expressing appropriately favorable “tumor immune profiles” (27, 34, 46, 47). These clinical observations support the existence of specific and permissive tumor qualities, immune molecules, and host responses that collectively contribute to therapeutic outcomes by modulating the interplay among the tumor, TME, and host. Seemingly similar tumors often respond in disparate manners to identical immunotherapeutic interventions, and these observations support the existence of heterogeneous immune compositions across tumors of similar histology (31, 34). The immune profile of a tumor is the result of combinatorial interactions between both intrinsic and extrinsic factors (31, 37), and recognition of these complex relationships can afford opportunity to rationally institute therapeutic interventions.

Cancer is a genetic disease, and the genome and epigenome of individual tumors can participate in generating both positive and negative immunosurveillance outcomes (31). Genomic instability early in the oncogenic process favors the acquisition of truncal driver mutations shared by a large fraction of clonal progeny tumor cells and can result in the expression of tumor-specific neoantigen targetable by immunotherapeutic interventions such as CAR-T cells. Conversely, genomic mutations can favor immunosuppression too, such as MAP kinase pathway perturbations that reduce MHC class I molecule expressions, with consequent immune tolerance within the TME. Complementing genomic alterations, gene transcription can be perturbed by epigenetic modifications, microRNAs, and non-coding long RNAs, leading to gene transcription of immunosuppressive molecules such as PD-L1, IDO-1, and IDO-2, as well as increasing suppressive cytokine secretion (31). Not unexpectedly, the genetic makeup and/or transcriptional regulation manifested by individual tumors can substantively contribute to the polarization of immune phenotypes, being either “hot” or “cold”.

In addition to genomic regulation, a multitude of additional factors contribute to shaping tumor immune profiles including the influences of the microbiome, tumor location, myeloid derived suppressor cells, light exposure, tumor stromal thickness, and type 1 interferon production (31, 37). In the case of the microbiome, the specific genus, Bifidobacterium, has been demonstrated to positively influence the quantity and/or quality of tumor-specific CD8^+^ T cells within the TME sufficient enough to delay tumor growth (48, 49). Another influencing factor involves myeloid derived suppressor cells (MDSCs) that develop from myelopoiesis during episodes of sustained inflammation and are phenotypically similar but functionally distinct from neutrophils and monocytes. MDSCs exert counterregulatory activities to impede exuberant T cell responses and can contribute to non-inflamed immune phenotypes (50). In addition, the tissue of origin can also affect the tumor immune profile, as anatomic compartments (central nervous system, gastrointestinal tract) where inflammation can be exceptionally damaging are basally repressed by the liberation of immunosuppressive cytokines. As an example, in colorectal cancer there tends to be elevated levels of TGFβ, and consequently increased numbers of inducible Tregs which promote a non-inflamed tumor profile (51, 52).

Stimulation of type 1 interferon is essential for fostering immune-inflamed tumor phenotypes (53, 54). Type 1 interferon production is prominent in amplifying the immune response through coupling of the innate and adaptive immune systems. Cooperativity between innate and adaptive systems cultivates an environment conducive for sustaining TILs that result in tumor antigen specific cytotoxic effector functions and immunological memory for durable anticancer immune activities (55). The main mechanism for amplifying the innate immune system with type 1 interferon is through augmentation of antigen presenting function of dendritic cells, macrophages and other APC’s in the TME, with consequent priming of T cells with tumor antigens. Underscoring the importance of type 1 interferon signaling for adaptive immunity, knockout mouse studies have demonstrated impaired T cell priming towards tumor antigens and resultant adoption of non-inflamed tumor phenotypes (56).

Given the importance of type 1 interferon for promoting tumor-inflamed profiles, considerable interests have focused on the actionability of stimulating cellular receptors on immunocytes known as pattern recognition receptors (PRRs) with damage associated molecular patterns (DAMPs) produced from a specialized form of cancer cell apoptosis (57, 58). The specific programmed cell death pathway mediated by the release of DAMPs from dying cancer cells that heightens immunogenicity is termed immunogenic cell death (ICD) (57). Beginning with calreticulin (CALR), an abundant endoplasmic reticulum (ER) protein, ER stress induced by ICD inducing agents cause CALR to translocate to the plasma membrane of dying cancer cells (15, 57–62). Importantly, any interruption of this initial CALR translocation step, prunes the immunogenic anti-neoplastic response and curtails the effects to non-immunogenic cell death (57). Membranous CALR exposure act as strong “eat me signals” so that dendritic cells and macrophages possessing the CD91 receptor become engaged as participants in the cancer-immunity cycle through active phagocytosis of CALR expressing tumor cells (57, 59, 60, 63). As cancer cells die, autophagy-dependent ATP release acts as strong “find me signals” for dendritic cells and macrophages (57, 59). Upon ATP binding to the P2RX7 receptor on dendritic cells, IL-1β is released, which draws tumor specific T lymphocytes into the TME (57). The final hallmark of ICD is the post-apoptotic release of the non-histone protein, high mobility group box 1 (HMGB1) (57, 59, 60). Upon release from dying cancer cells, HMGB1 serves as a cognate activating ligand for Toll-like receptor 4 (TLR4) on cells of the innate immune system such as dendritic cells (57, 59). The successful binding of HMGB1 to TLR4 is a necessary trigger for the release of proinflammatory cytokines such as type 1 interferon into the TME (19, 57). In tumors that are deficient of TILs, the innate immune system can be activated in order to promote tumor antigen presentation to cells of the adaptive immune system, and the purposeful induction of ICD can promote the activation of immunocytes within immunologically cold tumors (57). Broadly, strategies that leverage ICD incorporate the use of defined exogenous stimuli (chemotherapy, radiation, hyperthermia, physical stressors) to elicit a specialized form of immunogenic, regulated cell death, which can activate and bridge the innate and adaptive immune systems to prime the TME with TILs and thus, transform “cold” into immunologically “hot” tumors.

## Canine Immunogenic Tumors

**Figure 2:**
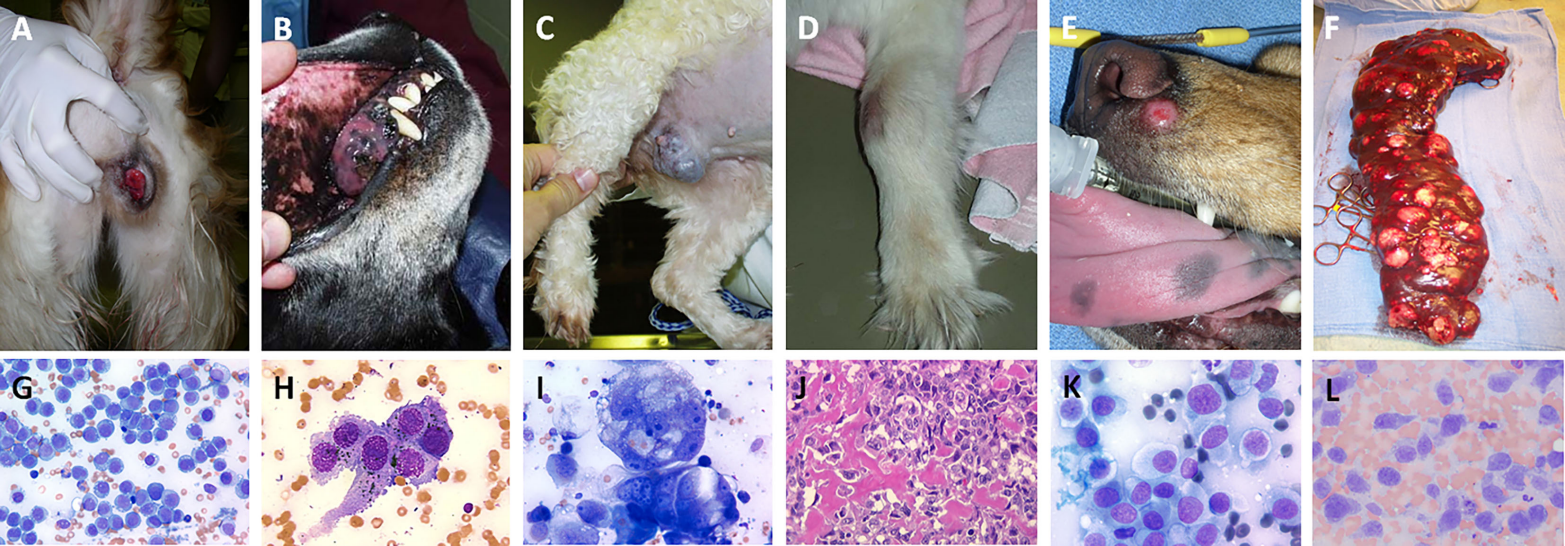
Panel of spontaneously-arising tumors in pet dogs demonstrating classic clinical presentation and associated representative cytologic or histologic features. All tumors presented are considered immunogenic in nature and can be leveraged for evaluation of novel immunomodulatory strategies for cancer therapy and include **(A, G)** canine transmissible venereal tumors, **(B, H)** oral malignant melanoma, **(C, I)** mammary gland carcinoma, **(D, J)** appendicular osteosarcoma, **(E, K)** cutaneous histiocytoma, and **(F, L)** splenic histiocytic sarcoma. Cytology and histology images, magnification 200-500x.

Immuno-oncology continues to cultivate significant promise in the world of cancer therapy, and as new immunotherapies are developed, it remains paramount to identify sophisticated tumor models for assessing the benefit and limitations of novel immune-activating strategies. Recognizing the distinct immune characteristics of different tumor histologies serve as an initial foundation to rationally design, tailor, and validate treatment effects which are intended to counteract breaks in the cancer-immunity cycle and enhance immunotherapy efficacy. As observed in human cancer patients, specific tumor histologies (melanoma, renal cell carcinoma, NSCLC, urothelial carcinoma, HNSCC) are considered more likely to be immunogenic (64), and better suited for evaluating novel combinatorial immunotherapeutic strategies. Analogously, in pet dogs, certain cancers are more immunogenic and understanding the foundational immune landscape of specific spontaneously arising tumors in pet dogs is necessary to serve as a natural reference point when evaluating the immune modulatory activities of investigational treatments. While not necessarily equivalent with translational relevance, six tumor histologies (**Figure 2**) including cutaneous histiocytoma, histiocytic sarcoma, transmissible venereal tumor, osteosarcoma, oral malignant melanoma, and canine mammary gland tumors will be highlighted for how the immune system participates in shaping the biology, clinical behavior, and prognosis of these tumors. With this foundation, specific tumor types of greatest comparative value can be uniquely leveraged to accelerate the discovery and validation of new immunotherapeutic interventions intended for human cancer patients.

### Cutaneous Histiocytoma

Canine cutaneous histiocytoma is classified as a benign epidermal tumor of Langerhan cell origin most often affecting young dogs less than 3 years of age (65–68). Spontaneous regression, typically within 2-3 months of presentation, is a unique and compelling feature of this tumor which can provide insights into naturally-occurring successful anti-tumor immune responses (67, 69). Upon presentation, histiocytomas often appear similar to other round cell tumors and immune molecular markers are nearly synonymous to other dendritic cell diseases (70, 71). Immunophenotypic markers of canine cutaneous histiocytoma including CD1a, CD11c, CD18, MHC class II, and E-cadherin (67, 68, 70, 71). Importantly, the expression of E-cadherin is a distinguishing adhesion marker indicating Langerhans cell origin (70), however cannot be used solely to diagnose cutaneous histiocytoma given overlapping expression by other round cell tumors (72).

A complete list of elements participating in the spontaneous regression of cutaneous histiocytomas remain unspecified (71); however, confirmed factors actively contributing to regression are mediated through anti-tumor responses by CD8^+^ αβ T cells (67, 71, 73). The initiation of T cell activation involves the migration of tumor derived dendritic cells, as well as infiltrating interstitial dendritic cells, to regional lymph nodes. Once in the draining lymph node, these dendritic cells engage CD4^+^ T lymphocytes which potentiate CD8^+^ T cell activation (71). It has been proposed and not discounted that the origin of cutaneous histiocytoma being the Langerhan cell, may contribute to a self-perpetuated mediated regression due to the normal antigen presentation function of dendritic cells (67, 71). This putative regressive mechanism, while not proven, remains plausible given that suboptimal antigenicity is a fundamental feature of failed immunosurveillance (74).

To better elucidate factors associated with spontaneous regression of cutaneous histiocytomas, a correlation between the magnitude of lymphocytic infiltration and dynamics of spontaneous regression has been studied (67). This study observed that as histiocytomas longitudinally proceed through later points of spontaneous regression, lymphocytic infiltration increases, as well as the transcription of pro-inflammatory molecules including IL-2, TNF-α, IFN-γ, and iNOS. By categorically segregating 30 histiocytomas into 4 groups based on stage of regression, early in the regression period, CD4^+^ T cells out number CD8^+^ T cells, but as tumor regression proceeded, CD8^+^ T cells comprised the predominant immune cell infiltrate. In a complementary study, canine cutaneous histiocytomas that spontaneously regressed displayed increased T and B lymphocyte infiltrates, decreased mitotic index, MHC class II molecule plasma membrane localization, and minimized E-cadherin expression on tumor cells (70). The loss of E-cadherin in normal dendritic cells following their activation allows them to travel to draining lymph nodes to prime cells of the adaptive immune system (70, 72), and this mechanism was postulated as a reason for loss of E-cadherin expression in spontaneously regressing cutaneous histiocytoma.

Typical treatment for canine cutaneous histiocytoma involves surgical excision if spontaneous regression fails to occur within 3 months of tumor presentation (66). Although infrequent, some cutaneous histiocytomas do not spontaneously regress and lymphadenopathy may ensue consequent to migration of tumorous histiocytes to draining lymph nodes (68, 71). In these progressive settings, it is probable that the cancer immune response is insufficient and offers opportunities to investigate strategies to manipulate the cancer-immunity cycle for reinduction of effective immune surveillance.

### Histiocytic Sarcoma

Histiocytic sarcoma (HS) is a highly aggressive tumor arising from interstitial dendritic cell origin and shares many similarities between human and canine patients (75). Although far less common in humans (76, 77), commonalities of genetic and biologic importance support canine HS a compelling comparative model for investigating its human counterpart (78). Fortuitously, canine HS is a relatively common tumor histology that arises spontaneously with enriched frequency in specific dog breeds such as the flat coated retriever, Bernese mountain dog, golden retriever, and rottweiler (68, 75). Of significant relevance, recent studies have demonstrated a prominent TIL composition in both human and canine HSs underscoring the existence of dynamic immune trafficking within the TME and hence the rational exploration of immunotherapies in both species.

In people, HS is universally aggressive in nature and strong clinical justification exists for improving disease management. Yet given its rarity in humans, definitive standard of care for HS remains loosely established, and further underscores the potential value of comparative models to accelerate the validation of investigational treatment options. Histiocytic malignancies in pet dogs can serve as a relevant translational model for human HS based upon conserved disease-associated genetic abnormalities and aggressive biologic behaviors. Through detailed comparative cytogenetic studies, the most recurrent DNA copy number aberrations (CNAs) identified in HS derived from flat coated retrievers and Bernese mountain dogs are evolutionarily conserved with those reported in human histiocytic malignancies too (78, 79).

Both human and canine HS are often associated with poor outcomes despite treatment, highlighting the need for novel therapeutic protocols. A recent study, documenting the immune makeup of visceral and localized HS in flat coated retriever dogs demonstrated that 95% of infiltrating T cells were positive for FOXP3 suggesting a high proportion of Treg cells within these tumors (80). In addition, CD45RA (naïve T-cell and hematopoietic marker) stained positively in a higher proportion of visceral canine HS of the spleen, while FOXP3 was increased in localized soft tissue HS. While not designed for definitive correlative analysis, this study did identify marginally improved outcomes in pet dogs with localized HS despite having a higher density of immunosuppressive Treg cells (80). Complementing these findings, a recent report characterizing the immune infiltration status with prognosis has been reported in both human and canine HS. Findings from this comparative study identified improved clinical outcomes in pet dogs with primary tumors demonstrating higher densities TILs expressing CD3^+^ and granzyme B (81). Counterintuitively, this study also found that increased levels of FOXP3 was associated with improved outcomes, although this relationship was not statistically significant (81). Additionally, this study found that immune infiltration and tumor location were the strongest prognostic indicators and highlight the potential influence of immune reactivity with positive treatment outcomes.

### Transmissible Venereal Tumor

Canine transmissible venereal tumor (TVT) circulates amongst both domestic and wild canid species and is proposed to be the oldest known cancer in existence, likely originating in a dog or wolf 10,000 years ago (82). Transmissible venereal tumor propagates across canids as a contagious cancer that resides as an allograft within affected individuals by circumventing host versus graft rejection typically mediated by major histocompatibility (MHC) protective mechanisms (82–84). Horizontal transmission, primarily through coitus, has allowed the perpetuation of the founding TVT cell lineage to be preserved from its initial emergence, and has spread globally as sub-clones from the original tumor (85). Immunohistochemical characterization of TVT supports a cancer of histiocytic cellular origin based on positive markers for vimentin, lysozyme, ACM1, and alpha-1-antitrypsin (82, 83, 86). In most cases, TVT is non-lethal and is characterized by three defined evolving phases, being the initial progressive (P) phase, leading to the stationary (S) phase, and ending with the regressive (R) phase in which spontaneous immune-mediated regression can occur (83). The progressive (P) phase typically lasts between 3-4 months in duration, while the stationary (S) phase can last for months to years depending upon multiple factors including immuno-competency (83). Spontaneous regression (R phase) can occur follow the waning of the (S) phase. In general, TVT behaves as a local malignancy and disseminated metastases occurs rarely (< 5%) (82, 87, 88), except in immunocompromised canids or puppies with ill adapted immune systems (83, 86).

While ultimately immunogenic and possessing potential to undergo spontaneous regression, TVT has the unique ability to avoid host immune rejection upon transmission as a cellular allograft (82, 83, 87). Typically, allografts should be rejected because of functional MHC restrictive barriers between individuals (82). However, TVT employs an immuno-suppressive mechanism largely reliant on the secretion of TGFβ1 by tumor cells (82, 87). Early during the (P) phase, an extreme scarcity of MHC class I and II molecules is expressed by tumor cells (< 5%) (82–84). In the absence of MHC molecules, TVT cells do not present sufficient quantity of tumor peptides in the context of MHC molecules, and can remain largely unrecognized by cytotoxic T cells (89). Additionally, the active secretion of TGFβ1 by TVT cells creates a hostile TME that impairs the proliferation of B and T cells, blunts cytotoxicity of T cells by inhibiting release of perforin and granzymes, blocks cytolytic activity of NK cells and bars the effects of IFN-γ induced expression of MHC class I and II molecules (89). Normally NK cells can kill cells that do not express MHC molecules, but TVT-derived TGFβ1 inhibits NK cell killing and permits TVT establishment and growth largely unabated during the (P) phase.

Spontaneous regression that occurs during the (R) phase of TVT is associated with a notable increase in MHC class I and II molecule expressions on tumor cells to 30-40% (82, 84, 89, 90). Nonetheless, the majority (60%) of TVT cells remain MHC barren and necessitate the reactivation of NK cells to regain their cytotoxic functions. Collectively, augmented MHC expression and restoration of NK cell cytotoxicity serve as driving immunostimulatory mechanisms responsible for TVT regression. Synergizing with cellular alterations favoring regression, IL-6 secretion by TILs emerges as a vital immunostimulatory cytokine and helps tip the (P) phase into the (R) phase (84, 89). Interestingly, IL-6 seems to be secreted by TILs throughout both (P) and (R) phases of TVT cellular lifecycle; however, during the progressive stage, IL-6 secretion is low, while in the regressive phase it is much higher (89). IL-6 promotes immune recognition and attack of TVT cells by counteracting TGFβ1 activity and thereby attenuating TME immunosuppression (89, 91). Additionally, IL-6 can reestablish IFN-γ activity to amplify MHC class I expressions (84). In summary, the interplay between MHC expressions, IL-6, IFN-γ, and TGFβ1 appear to be major participants involved in the (P) phase and (R) phase of TVT. Complementing these observed cellular immune dynamics, there is evidence suggesting humoral responses to TVT might likewise participate in immuno-surveillance, as supported by the detection of IgG antibodies against a tumor specific antigen (92). Humoral protection of detected antibodies appears functional, as the passive transfer of antibodies from previously infected dams has been shown to be capable of establishing resistance to TVT in nursing puppies (92).

Given the contributory role of TGFβ1 in TVT pathogenesis and immune evasion, pet dogs with TVT could serve as a valuable model system for evaluating that tolerability and immune reactivating potential of TGFβ1 inhibition strategies, which include blocking antibodies, ligand traps, decoy receptors, and small molecule inhibitors (93). The near-term opportunity to evaluate TGFβ1 inhibition strategies in pet dogs has been accelerated by studies describing key tissue tolerability of small molecule inhibitors (galunisertib) in beagles (94), as well as the *in vitro* biologic activity of novel fusion proteins in canine cell lines (95). However, given the pleiotropic activities mediated by TGFβ1 beyond immune suppression, deconvolution of provocative results generated in pet dogs with TVT would be required, given TGFβ1’s central roles in angiogenesis and epithelial-mesenchymal transition.

### Osteosarcoma

Osteosarcoma (OS) is the most common primary bone tumor in dogs; accounting for up to 85% of malignancies originating from the skeletal system and affecting more than 10,000 dogs per year in the United States (96, 97). Canine OS closely resembles pediatric OS with regards to histological features, spontaneous development, predilection for larger skeletal size, molecular signatures, high instance of aneuploidy, metaphyseal and appendicular localization tendency, high metastatic potential, and aggressive nature (98, 99). Based upon the strong conservation of OS biology, pet dogs provide an exceptional comparative model for human OS. Middle-aged to older dogs that are large or giant breed are most commonly affected (100, 101). Due to its high metastatic potential, it is estimated that 90% of canines will have established micro-metastasis at initial presentation, and dogs treated with amputation alone succumb to metastatic progression within 20 weeks (102). Canines treated with standard-of-care, inclusive of amputation and systemic chemotherapy have improved survival times approaching 9-11 months (103, 104).

Canine OS is considered an immunogenic tumor based upon serendipitous and purposeful scientific inquiry, and has been thoroughly discussed in recent review publications highlighting historical and active evidence of OS immune recognition (13, 105–107). While there has been convincing clinical evidence for canine OS immunogenicity derived from the antimetastatic activities exerted by L-MTP-PE and inhaled liposomal IL-2 (108–110), as well as, retrospective observations for improved survival outcomes in pet dogs developing limb-spare infections (111, 112), only recently has there been a focused attention on the exact cellular players that might be orchestrating provocative antimetastatic activities through heightened immunosurveillance.

Much of what is known about the immune response in OS and associated prognostic relevance implicates monocytes, macrophages, and regulatory FOXP3^+^ T (Treg) cells. Of note, FOXP3, although often used to identify Treg cells, may also be expressed in other subsets of T cells. Pointedly, macrophages have been emphasized as main contributors towards improving overall survival time and disease-free interval in canines as well as humans. Supporting the importance of macrophages, Withers et al. performed a study to characterize lymphocyte (CD3^+^ and FOXP3^+^) and macrophage (CD204^+^) infiltrates within the OS TME and their effects on disease free interval and overall survival time (113). The study involved 30 dogs treated with standard-of-care defined as surgical amputation and carboplatin therapy. Pet dogs with a greater percentage (≥ 4.7%) of cellular infiltrates being CD204^+^ within the primary tumor and associated TME achieved markedly enhanced disease-free intervals. Lymphocytic infiltration, regardless of presumed effector or regulatory phenotype, was not associated with outcomes. While increased mononuclear infiltrate within the primary tumor appears to support improved immunosurveillance, this supposition has not been supported with differences in circulating immunocytes, whereby elevated numbers of circulating monocytes (>400 cells/µL) and lymphocytes (>1000 cells/µL) were associated with decreased disease free intervals (114). To further describe potential functional changes of circulating monocytes in dogs with OS, Tuohy et al. identified altered and reduced monocyte surface expression of several chemokine receptors (CD62L, CCR2, CCR7, CD43, CX_3_CR1, and CXCR2) in dogs with OS versus healthy dogs (115). Based upon these observational findings, it was postulated that downregulation of these chemokine receptors imparted a functional reduction in the directional migration of monocyte into the primary tumor or metastatic sites (115). While not proven by either study characterizing monocyte populations within circulation, it could be speculated that dysfunctional monocyte/macrophage migration could result in breaks in *Steps 2 & 3* (antigen presentation and secondary lymphoid tissue migration) of the cancer-immunity cycle, and thus reduce effective anticancer immunity for combatting metastatic progression.

In addition to the role of monocytes and macrophages for OS immunosurveillance, absolute and relative lymphocyte phenotypes (effector versus regulatory) have been investigated in both human and canine OS. In humans, considerable evidence supports the negative immunoregulatory activity of FOXP3^+^ Tregs in solid cancers, including pediatric OS. In a recent investigation utilizing 150 treatment-naïve patient biopsies, researchers demonstrated that intratumoral CD8^+^/FOXP3^+^ ratio in the OS microenvironment is predictive for survival (116). Similarly, a study in canine OS patients evaluating CD8^+^/FOXP3^+^ ratios in blood, lymph node, and primary tumor also concluded comparable findings, being dogs with decreased CD8^+^/FOXP3^+^ ratios were associated with significantly shorter survival times (117). Lastly, increasing evidence indicate that canine OS possess the cellular machinery to attenuate effector T cell attack, specifically tumoral PD-L1 expressions (118–120), and could ultimately promote T cell exhaustion. Derived from these early studies, PD-L1 is likely taking part in the immune evasion and suppression mechanisms, as supported by the inverse relationship between OS PD-L1 expression and the number of intratumoral T cells (118).

### Oral Malignant Melanoma

Canine oral malignant melanoma (OMM) is considered a highly aggressive tumor that is locally invasive and highly metastatic. In dogs, OMM accounts for 40% of all oral cancers and carries a guarded prognosis for long term survival (121). Traditional therapies are reliant upon local and distant tumor control requiring multimodality treatment approaches including surgery, radiation, and chemotherapy; however, outcomes for disease management are often non-curative in nature. While the management of primary disease within the oral cavity can be adequately controlled with surgery and/or radiation (122, 123), the effective control of regional and distant metastases remains problematic even with the institution of systemic chemotherapies (124). There is strong evidence that canine OMM can serve as a valuable comparative tumor for improving treatment of advanced melanoma in human beings given the shared clinical behavior, genetics, and disease biology between species (125, 126). Of significance, recent metastasis-associated gene expression profiles and comparative transcriptomic analysis have become available for canine OMM (127–129), and provides solid genomic foundation for leveraging a comparative oncology approach for validating new therapies to curb metastatic progression in both canines and human beings. Excitingly, like the tremendous opportunity already realized for remarkable immunotherapeutic outcomes in people with melanoma treated with checkpoint blockade strategies, there is rapidly accumulating data to suggest parallel outcomes are also feasible in dogs (130–132), and solidifies canine OMM as an outstanding comparative model to explore novel immunotherapeutic combinations intended to benefit people diagnosed with advanced melanoma (cutaneous and mucosal) (133).

Despite the strong evidence supporting the comparative nature of canine OMM as a model for human melanoma, it is important to call attention to a few relevant distinctions. Human malignant melanoma is often cutaneous and is frequently UV induced creating a high mutational burden, and thus, a high level of immunogenicity. Although canine OMM is not thought to be associated with UV induction, it remains immunogenic with the potential production of neoantigens which can be recognized by the immune system. Confirmation of putative OMM immunogenicity has been supported by a recent study evaluating tumor mutational burden (TMB) across 7 tumor types in over 35 canine breeds (134). Within the 7 tumor types evaluated, canine OMM, along with OS and hemangiosarcoma, were among the cancer types having the greatest amount of TMB. Interestingly and similar to humans, the TMB across all tumor types and breeds was frequently associated with mutation of the tumor suppressor gene, TP53. Furthermore, canine OMM demonstrated similar pathway alterations analogous to human melanoma, in particular p53 and cell cycle alterations. Collectively, this study indicated that TMB tracked primarily with tumor type, and breed was not found to be a driving factor. Further evidence supporting the immunogenicity of canine OMM has been demonstrated in a study showing that across three forms of canine melanoma, a high percentage (68%) exhibited presence of TILs within the TME. Similar to human cutaneous melanoma, the overall abundance of TILs was assessed as mild. In addition, a higher proportion of CD20^+^ B lymphocytes was associated with an increased occurrence of metastasis and tumor related death compared to elevated tumor infiltration of CD3^+^ T lymphocytes (135). Taken together, while the underlying carcinogenic factors that drive melanoma formation in dogs and people might be divergent, there remains significant overlap in regard to tumor immunogenicity in both canines and people. Given this immunologic conservation, canine OMM should be considered a valid comparative model for evaluating novel immunomodulatory strategies that have potential to improve treatment outcomes in human beings.

Canine OMM is a highly immunogenic tumor, and cancer vaccination strategies has garnered attention over the past 2 decades for their potential to improve prognosis for dogs diagnosed with OMM (136, 137). While multiple vaccination strategies have been explored including genetically modified allogenic cell vaccination (138), a xenogeneic DNA tyrosinase canine vaccine has been widely used clinically since 2007, and has demonstrated in some studies to enhance survival times in dogs with OMM (139, 140). In one study comparing historical outcomes of 53 dogs diagnosed with OMM that were not treated with the human tyrosinase vaccine to 58 dogs with stage II or III OMM that received the human tyrosinase vaccine, a significant survival benefit was identified in dogs receiving vaccine treatment (140). Despite these positive findings, other retrospective investigations analyzing the efficacy of human tyrosinase vaccine in the adjuvant setting for canine OMM have not identify significant improvement in progression-free survival, disease free interval, or median survival time for those animals treated with tyrosinase vaccination (141, 142). Currently, there are several investigations demonstrating the safety and biologic outcomes associated with human tyrosinase vaccination in dogs with varying clinical stages of OMM (stages I-IV), but few of significant population size (143). While the immunogenicity of OMM is not disputed, collectively, the findings derived from these existent reports with human tyrosinase vaccination would be strengthened through the conductance of prospective definitive trials with appropriate control arms.

Complementing human tyrosinase vaccine approaches, some investigations have explored parallel vaccine strategies utilizing different xenogeneic proteins as immunogens. One such strategy includes intramuscular electrovaccination of a DNA plasmid encoding human chondroitin sulfate proteoglycan-4 (CSPG4) into dogs with OMM (144, 145). In the first foundational study, CSPG4 electrovaccination was safe, improved disease-free intervals in vaccinated compared to unvaccinated dogs, and generated measurable immune responses assessed by antibody production and IFN-γ T cell responses (144). In a follow up prospective study, electrovaccination of human DNA plasmid for CSPG4 was evaluated in the adjuvant setting following surgical resection of primary OMM lesions in 23 dogs. The median disease-free interval, median survival time and 1- and 2-year survival rates were compared to a group of 19 dogs treated with surgical resection only. The experimental group receiving CSPG4 adjuvant vaccination achieved significantly improved outcomes compared to the non-vaccinated group (145), and these results further support the potential clinical value of CSPG4 vaccine strategies. Further bolstering the promise of vaccine approaches for improving OMM outcomes, a pilot study employing the intratumoral delivery of an adenovector CD40L transfection strategy has been conducted in dogs with malignant melanoma, and following vaccination, resected melanoma tissues had B and T lymphocytes trafficking through the tumor parenchyma indicating the existence of an immune-inflamed profile (146).

In addition to vaccines which generate adaptive and protective immunity through cell-mediated activities, monoclonal antibodies have revolutionized cancer therapies for both hematopoietic and solid tumor histologies in people (147–149). While the realization of monoclonal antibodies in veterinary medicine remain largely unrealized for cancer applications, tremendous opportunity exists for systemically exploring combinatorial immunotherapeutic strategies inclusive of checkpoint blockade in dogs with OMM. Historical and active investigations continue to indicate that naturally-occurring tumor histologies, in particular OMM, might be responsive to checkpoint blockade interventions (PD-1/PD-L1) (120); with predicted reversal of exhausted T cell responses following checkpoint inhibition being predicated on existing data that demonstrates an immune-inflamed phenotype of OMM (135, 150), in conjunction with the expression of PD-L1 by OMM cells (120, 130, 132).

The druggability of the PD-1/PD-L1 axis in OMM has been developed over the past 5-10 years and continues to advance with provocative translational potential. In early studies performed by Maekawa et al., IFN-γ induced expression of PD-L1 on immortalized canine melanoma cell lines, and endogenous expression of PD-L1 was confirmed in naturally-occurring OMM tissue samples (47). Further characterization of PD-L1 expressions performed by Maekawa et al. reaffirmed high PD-L1 expression on 90% (36/40) of canine OMM samples (120). Furthermore, this study confirmed PD-1 expression on both CD4^+^ and CD8^+^ TILs, and illustrated how the cancer-immunity cycle could be short circuited through OMM PD-L1 expressions to promote T cell exhaustion (120). The functionality of OMM-mediated PD-L1/PD-1 immunosuppression was further supported by a complementary investigation that evaluated the immunobiologic activities of an anti-PD-L1 antibody administered to dogs with OMM, showing increased survival time in 4 dogs with pulmonary metastasis compared to a historical group (132). In a most recent investigation, the safety and efficacy of a canine chimeric anti-PD-L1 monoclonal antibody (c4G12) administered intravenously every 2 weeks was assessed in 29 dogs with advanced OMM and pulmonary metastases. In a subset of 13 dogs with measurable pulmonary metastatic disease, single agent activity was documented in 1 patient achieving complete remission, with a calculated overall response rate of 7.7% (1/13) (130).

While single agent activity of anti-PD-L1 blockade appears modest in the setting of advanced pulmonary metastatic OMM (130), combinatorial adjuvant treatment option for use in patients with OMM that should be aggressive explored with the expectation for developing rational combinations capable of exerting additive and/or synergistic activities. Such innovative strategies should include the therapeutic induction of ICD which are predicted to generate abscopal effects when induced by ionizing radiation in canine OMM patients (15), as well as other innovative approaches to overcome existent breaks in the cancer-immunity cycle.

### Mammary Gland Tumors

Canine mammary gland tumors (MGTs) are the most common cancers diagnosed worldwide and predominantly arise in sexually intact female dogs (151). Middle-aged to older dogs are at higher risk for developing MGTs, while in dogs under 5 years of age, the risk of developing aggressive MGT with associated metastatic potential is lower (152, 153). Given the hormone-dependent nature of mammary gland epithelium, the risk of tumor development is significantly reduced in dogs undergoing ovariohysterectomy prior to onset of the first estrus cycle; and dogs spayed before the first estrus cycle have only a 0.5% lifelong risk of developing MGTs (154). In addition to hormonal status, other risk factors for MGT development include breed and body condition status during puberty (9-12 months) (155). Similar to other cancers, genomic instability is believed to play a role in MGT tumorigenesis, and evidence suggests mutations in the *BRCA* genes might serve as risk factors in canine MGT development, but this supposition requires further investigation (156, 157).

The immune system imparts elements of risk or protection to dogs diagnosed with MGTs as well, and lymphocytes are the dominant inflammatory cell type infiltrating canine mammary gland tumors (158–161). In some studies, the pattern and quantity of TILs have provided prognostic information. As one example, Carvalho et al. identified risk factors related to the specific location of T lymphocytes within or surrounding the TME that showed significant relationships to survival (159). In benign tumors, there were higher numbers of CD3^+^ TILs compared to malignant tumors, yet in malignant tumors a greater density of CD3^+^ lymphocytes were constrained to the tumor periphery. These microanatomic findings could support that malignant MGT can adopt immune-excluded phenotypes, and consequently evade immunosurveillance and tumor progression is not constrained by the immunity-cancer cycle. Collectively, these findings provide some observational evidence that T lymphocyte location (intratumoral versus peripheral stroma) and quantity could be participating in MGT biology and behavior.

The notion that T lymphocytes can exert immune activities which affect MGT progression is not surprising, as T lymphocytes can differentiate into specific effector/regulatory phenotypes and consequently influence their microenvironment through both direct cell contact and soluble factors (162). In both human and canine MGTs, an elevated tumoral CD8^+^ T cell presence has been associated with a better prognosis and a decreased chance of metastatic progression (158). Conversely, a high level of CD4^+^ T cells within the TME can be a poor prognostic indicator in both human and canine MGTs. Because naïve CD4^+^ T cells can be polarized to discrete subtypes following activation including Th1, Th2, Th9, Th17, and T regulatory (Treg), further characterization of CD4^+^ T cells found within the MGT TME would more accurately represent key T lymphocyte compositions that contribute to MGT behaviors. Polarization of CD4^+^ subsets can have divergent immunomodulatory effects with the TME, with Th1 cell responses being pro-inflammatory in nature with the release of IFN-γ, TNF-α, and IL-2, while Th2 cell responses can be more suppressive through the release of IL-4, IL-5, IL-10, and IL-13.

In a study performed by Kim et al., investigators aimed to better characterize TIL local cytokine production and the role inflammatory cytokines on canine MGT progression (163). T lymphocyte secretion of IL-1 was associated with malignant and metastatic canine MGTs, potentially because IL-1 can mediate tumor cell proliferation and increases angiogenesis indirectly by promoting inflammation. Another inflammatory cytokine, IL-6, has also been investigated and was found to be secreted by canine MGT cells and significantly elevated in malignant and metastatic tumors compared to benign tumors indicating a possible pro-tumorigenic role (163). Finally, TNF-α was elevated in all tumor types but was the highest in malignant MGTs compared to benign and metastatic lesions. Normally TNF-α serves as an effector molecule capable of exerting anti-tumor immune effects through direct cancer cell killing, however TNF-α can also exert pro-tumorigenic activities in tumor cells and could be an underlying reason for observing elevated TNF-α levels in malignant MGTs.

Extending studies beyond classic Th1 and Th2 lymphocyte polarization, additional studies have implicated IL-17 in canine MGT malignancy, progression, and metastases (158); findings that are comparable to human breast cancer studies showing IL-17 to be a negative biomarker for prognosis and shorter disease-free intervals (164). As a counterbalance to immune inflammation, the participation of Treg cells in canine MGT has likewise been investigated for their ability to suppress anti-cancer immunity. In canine MGT, higher numbers of Treg cells within the TME have been associated with more aggressive forms of this cancer and poorer prognosis (165). In aggregate from these descriptive studies in canine MGT, it can be stated that multiple T lymphocyte subtypes can infiltrate into the tumor parenchyma and associated TME, yet true functional investigations are lacking and bar deeper interrogation for how the composition and dynamics of T lymphocytes might shape the biologic behavior and immunogenicity of canine MGT.

In addition to immunomodulation, the angiogenic effects of TILs can powerfully shape the TME, and provide plausible explanation for why elevated quantities of infiltrating CD3^+^ T lymphocytes have been correlated with worsened prognosis and decreased survival in both canine and human mammary tumors (160). One mechanism that should be recognized is TIL-induced angiogenesis. The ability of CD3^+^ T lymphocytes to secret inflammatory cytokines which consequently upregulate VEGF production and associated tumoral angiogenesis can contribute to the poorer prognosis and increased incidence of metastatic disease in MGTs with higher levels of tumor infiltrating CD3^+^ T cells (160, 166). Synergistically, certain T cell-secreted cytokines directly promote endothelial cell migration and proliferation, supplementing new blood vessel growth and heightened metastatic potential (166). The expression of other immune-related machinery, including chemokine receptors CXCR3 and CCR2, have been investigated for their participation in canine MGT biology. The expression of CXCR3 on malignant tumor cells has been correlated to increased tumor growth and spread (167), while CCR2 participates in tumor metastasis by promoting the trafficking of tumor associated macrophages into the TME with consequent angiogenesis (158).

The potential for canine MGT to induce T cell exhaustion through PD-L1 expressions has been described (120, 131, 168, 169), and findings support that canine MGT might employ this T cell suppressive mechanism to attenuate cancer cell killing. In addition to PD-L1, a recent study likewise identified the co-expression of CTLA-4 by CMT cells (168). Collectively, both PD-L1 and CTLA-4 tumoral expressions at gene and protein levels were found to be greater in metastatic MGT and were prognostic for survival time. Given these reports confirming PD-L1 and CTLA-4 expressions, checkpoint blockade therapy could be a promising treatment to enhance anti-cancer immunity in canine MGT patients. Furthermore, CTLA-4 binding to its ligand on tumor cells or antigen presenting cells enables T cell exhaustion. Peripheral blood T cells of dogs with neoplasia have demonstrated expression of CTLA-4, making this a potential therapeutic target in canine patients (170). Lastly, while great interest resides in checkpoint blockade strategies, canine MGT might also lend itself well to specific vaccine approaches given the recent description of melanoma associated antigen (MAGE-A) on canine MGT cells (171). MAGE-A consist of a group of tumor associated antigens, and are immunologically exploitable through diverse immunotherapeutic approaches including vaccines and transgenic T cell approaches (172, 173).

Canine MGTs are recognized by the immune system, with the quality, quantity, and composition of TILs influencing disease outcomes. The identification of immunocytes within the tumor parenchyma and associated TME affords the opportunity to modulate specific steps of the cancer-immunity cycle and potentially expand therapeutic options beyond traditional surgery, chemotherapy, and endocrine approaches. Given the similarities between human and canine MGT biologic behavior, pet dogs diagnosed with MGTs should be explored for their potential utility to advance immunotherapy practices for both of these species (166).

## Pet Dogs for Evaluating Cancer-Immunity Cycle Manipulations

**Figure 3:**
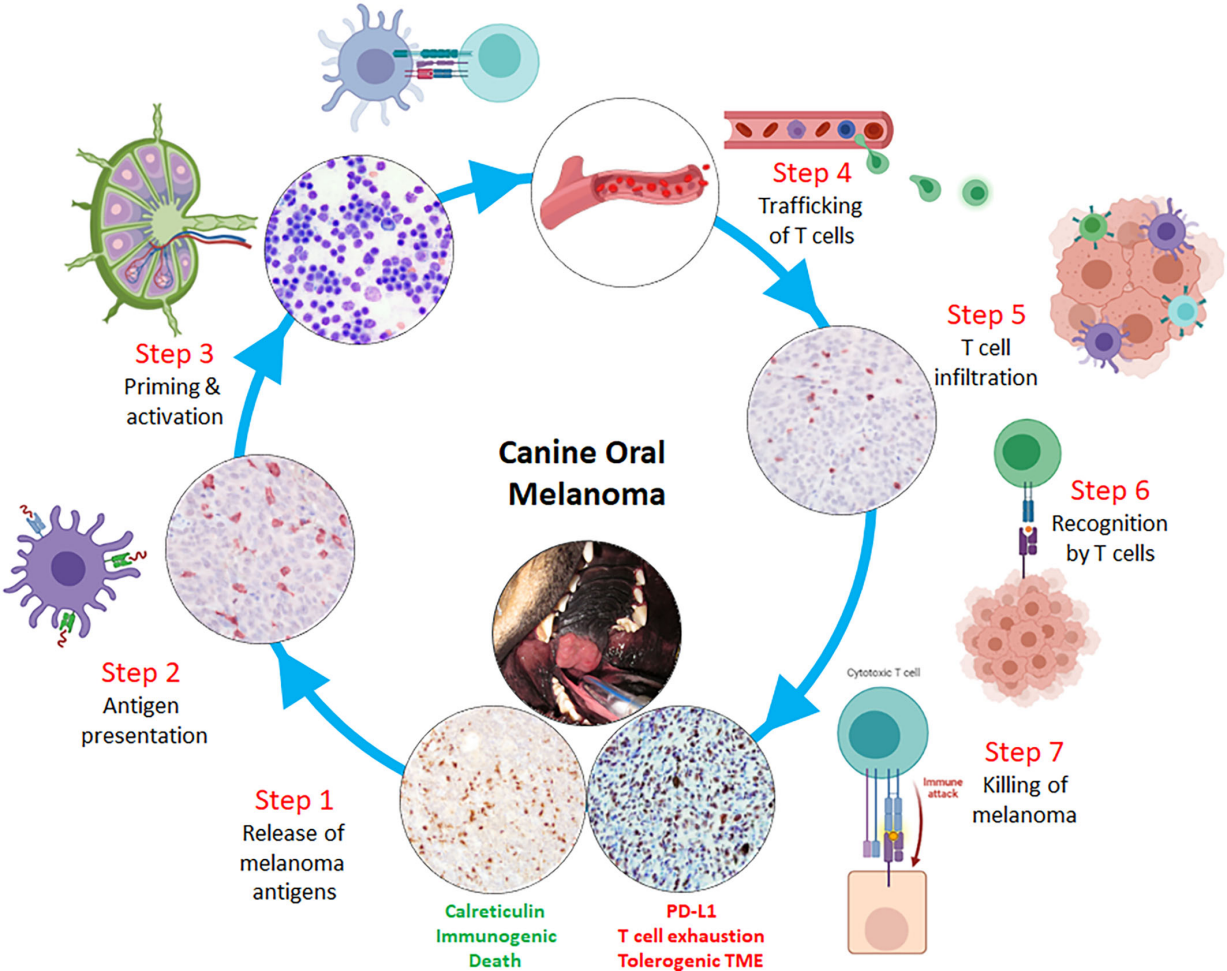
Rational design of immunotherapeutic strategies through a comparative oncology approach including canine oral malignant melanoma as a model tumor for correcting potential breaks in the cancer-immunity cycle. Sequential or combinatorial interventions can be evaluated at each potential break within the cycle, starting with: ***Step 1***- release of melanoma antigens following immunogenic cell death; ***Step 2***- sufficient intratumoral dendritic cell/macrophage processing and presentation of melanoma antigens with subsequent migration to secondary lymphoid organs; ***Step 3***- effective priming and activation of naïve T lymphocytes; ***Step 4***- intravasation and extravasation of activated T lymphocytes through hematogenous and/or lymphatic routes; ***Step 5***- sufficient T lymphocyte infiltration through microenvironment and into melanoma parenchyma; ***Step 6***- T cell receptor engagement with melanoma MHC class I:peptides; and ***Step 7***- effector T lymphocyte killing of melanoma cells.

Manipulating steps in the cancer-immunity cycle with immunotherapies that potentiate the cycle’s normal activities is a lucid approach to justify rational treatment interventions (**Figure 3**). Beginning with *Steps 1-3*, failure to produce actionable neoantigens diminishes the chances of professional antigen presenting cells (APCs) from successfully exhibiting altered-self and/or reactive antigens to naive T cells, ultimately resulting in an uninflamed TME. One way to override these initial, yet critical, immunologic barriers is through the delivery of tumor vaccines. Vaccines bolster antitumor immune responses through the purposeful administration of tumor-associated antigens with adjuvants, which will be taken up by APCs. These activated APCs will consequently migrate to lymphoid tissues, seek out reactive T cells, and consequently elicit a tumor-specific, cell-mediated response. Currently, there are numerous vaccine strategies including allogeneic whole-tumor cell vaccines, dendritic cell vaccination, and DNA vaccine approaches (174). In pet dogs with cancer, specific vaccine strategies have been investigated and provide unique opportunities to define the tolerability, effectiveness, and limitations of particular interventions to amplify *Steps 1-3* of the cancer-immunity cycle.

To date, most investigational vaccine strategies have been trialed in pet dogs diagnosed with metastatic solid tumor histologies including OS or OMM. For canine OS, a vaccine (ADXS31-164), comprised of a highly attenuated, recombinant *Listeria monocytogenes* expressing a chimeric human HER2/neu construct, has shown provocative activity for delaying micrometastatic disease progression when used as an adjuvant therapy following limb amputation and chemotherapy (175). However, despite being genetically attenuated, the use of live *Listeria monocytogenes* as a gene vector has raised concern given its zoonotic potential, and a lyophilized formulation used in pet dogs was deemed unacceptable for continued clinical development given the isolation of live *Listeria* within bodily fluids and tissues from treated canine patients (176). Analogous to the targetable antigenicity of cutaneous melanoma in people, the immunogenicity of canine OMM has underpinned the development and therapeutic assessment of genetically modified vaccine approaches that exert reproducible and measurable immune activities in specific subsets of dogs achieving clinical benefit. Several immunogens have been explored to elicit anticancer responses, particularly xenogeneic proteins (138, 146), and a bacterial plasmid DNA vaccine encoding xenogeneic human tyrosinase (Oncept™) is currently approved by the USDA for the adjuvant treatment of stage II/III canine OMM (139, 140). The commercial availability of Oncept™ permits widescale availability of this therapeutic for assessing traditional [radioimmunotherapy (143)] and innovative combinatorial studies. While OS and OMM classically have been leveraged as translationally-relevant models for comparative immunogenicity trials, vaccinal approaches for non-solid tumor histologies (CD40-activated B cell cancer vaccine) and pan-cancer immunogens (canine DNA telomerase vaccine) have also been conducted with generation of data sufficient to stimulate future research studies (177, 178).

Another means to generate immunogenic antigens, and amplify *Steps 1-3* of the cancer-immunity cycle, is through the purposeful induction of immunogenic cell death (ICD) (32). Broadly, ICD involves the use of a defined agent that has the capacity to elicit a specialized form of cell death that unlike normal apoptosis, is immunogenic (57, 58, 179). The release of three primary damage associated molecular patterns (DAMPs) being membranous translocation of CALR, release of ATP and HMBG1, enables ICD to engage dendritic cell (DC) maturation which prompts DC migration to tumor draining lymph nodes, antigen presentation, and expression of required co-stimulatory molecules for T cell activation (57, 60, 180). Immune escape resulting in non-inflamed tumors is partially attributed to insufficient production of immunogenic cancer antigens, and the induction of ICD through different therapeutic modalities [chemotherapy, radiation, oncolytic viruses, photodynamic and hydrostatic stress, hyperthermia (181)] offers multiple synergistic avenues to restore the first critical *Steps 1-3* necessary for optimal cancer-immunity cycle propagation. Given the breadth of ICD inducing modalities (58), the inclusion of pet dogs with cancer to conduct rapid screening of combinatorial approaches could accelerate the derivation of the most optimal treatment protocols related to sequence, dosage, and timing.

In pet dogs with cancer that deviate from successful T cell priming and activation, preempting this break in the cancer-immunity cycle (*Steps 2-3*) through the use of exogenous cytokines such as IL-2 and IL-12 may create the desired pro-inflammatory milieu that substantiates proliferation and activation of T cells and NK cells. In dogs with cancer, the exploration of single agent IL-2 as an anti-tumor immune cytokine has been demonstrated by subcutaneous, intratumoral, inhalation (liposome), and intravenous (DNA plasmid) routes in several different tumor histologies such as OS, mast cell tumor, and peripheral nerve sheath tumors (108, 182–184). In aggregate, IL-2 has proven to be active in the context of immunogenic solid tumors, being OS, but failed to demonstrate clear clinical benefit in tumors of undetermined immunogenicity (peripheral nerve sheath tumors). Based upon promising single-agent activity in dogs with OS, IL-2 has been incorporated into more complex combinatorial regimens inclusive of autologous cell vaccine and adoptive T cell transfer, with generation of favorably results and justifies larger scale confirmatory studies (185). The exploration of IL-12 in pet dogs remains rudimentary and limited by its conserved toxicity across species. However, innovative strategies for targeting IL-12 that theoretically limits systemic leakage have been investigated through the inclusion of pet dogs with cancer (186), and serves as clear example for the unique biologic value that can be gleaned from a comparative oncology approach. Nonetheless, additional strategies for localizing IL-12 must be evaluated for improved safety and activity, and it should be expected that pet dogs can provide high value information to expedite the clinical translation of similar strategies in human cancer patients (187).

For defects in *Steps 5-6* of the cancer-immunity cycle, adoptive therapies can maximize the chances for ensuring a sufficient number of effector cells (cytotoxic T and/or NK) are present and can extravasate and infiltrate into tumor lesions. Adoptive T cell therapy, explicitly chimeric antigen receptor (CAR) T cells can be employed to ensure target recognition and effector activities against tumor-associated targets including cluster of differentiation markers (CD19, CD22) and have produced lifesaving outcomes for the treatment of leukemia and lymphoma patients in human medicine (188). However, the process of creating CAR T cells can be costly and time consuming making it less amenable to becoming standard-of-care in veterinary medicine. Nonetheless, proof-of-principle investigations have been conducted using canine derived materials demonstrating the feasible for engineering CAR T cells capable of targeting HER2 or CD20 expressed by canine OS or B-cell lymphoma cells, respectively (189–192). Translation of canine CAR T cell technology remains in its infancy with required optimization of lymphodepletion strategies to maximize CAR T cell engraftment and survival. However, a first-in-dog study has demonstrated glimmers of activity of CD20 CAR T cells against B-cell lymphoma in a small number of pet dogs (191). A complementary adoptive cell therapy includes the *ex vivo* expansion and reinfusion of NK cells (193). Unlike cytotoxic T cells that require recognition of peptide:MHC class I complex, NK cells are equipped to target and kill tumor cells that do not express MHC class I on their surface. An innovative treatment design combining radiation therapy and autologous NK transfer has been reported in dogs with OS, and results demonstrate the feasibility and alluring antimetastatic activities achievable in the absence of conventional chemotherapy (194). With the refinement of NK cell enrichment strategies, it is highly anticipated that this form of adoptive cellular therapy will contribute to revolutionizing treatment outcomes in metastatic solid tumors such as OS (195).

Even with the promise of adoptive cellular therapies, any treatment benefit might be transient if the TME is unfavorable for sustained effector activities (*Step 7*). One mechanism that has dominated the immunotherapeutic landscape is the role of checkpoints, which are natural compensatory brakes to mitigate unrelenting immune activation. However, tumor cells subvert this immune defervescence safeguard, essentially causing infiltrating T cells to become exhausted. Mechanistically, tumor cells evade T cell killing *via* upregulation of checkpoint molecules, in particular PD-L1 on tumor cells and PD-1 on T cells. Reversal of this checkpoint through blocking strategies inclusive of anti-PD-L1, -PD-1, and/or -CTLA-4 antibody therapy provides robust benefit to some patients with immune-inflamed tumors profiles. While response rates to single or combined checkpoint blockade frequently are less than 30% across diverse tumor histologies, some patients are cured from their cancer completely, indicating that for these outstanding responders the break in the cancer-immunity cycle resides in the final step of T cell killing.

Given the remarkable effectiveness of checkpoint blockade strategies in people, there has been clinical urgency to develop and evaluate similar therapeutic reagents for the treatment of pet dogs with diverse tumor histologies predicted to respond to checkpoint inhibition (47, 118, 120, 196, 197). While a commercially available canine PD-1 antibody remains in-development, several translationally impactful studies have emerged over the past 5 years describing *in vitro* biologic activity and *in vivo* therapeutic potential of anti-PD-1, anti-PD-L1, or other checkpoint blocking molecule strategies alone or in combination in cancer-bearing dogs (47, 120, 131, 132, 198, 199). In totality, these cutting-edge investigations demonstrate an early glimpse into the provocative activity that could be unleashed through checkpoint blockade strategies in pet dogs with solid tumors (OS, OMM, glioma, sarcoma). It would be expected that rather than being used as monotherapies, and informed by the cancer-immunity cycle, implementing precisely timed and sequenced combinatorial treatment protocols inclusive of checkpoint blocking antibodies would generate additive anticancer benefit to a broader swathe of patients.

## Conclusions

Intuitively, the inclusion of species that lie closely on the evolutionary scale are more likely to share orthologous genotypes and prove more valuable as model systems for the faithful recapitulation of disease states. This paradigm has been applied to the study of cancer across different species and is termed comparative oncology. While the foundations of comparative oncology have been rooted traditionally in more conventional vertebrate species such as zebrafish and rodents, the purposeful inclusion of large animal models with spontaneously-arising tumors, such as pet dogs and cats, has flowed into consideration for accelerating the advancement of novel cancer therapies. Given the natural development of cancers in companion animals with operative immunosurveillance, it is expected that shared tumor histologies between people and pets will abide by the same rules governing the cancer-immunity cycle and could both benefit from similar immunotherapeutic cancer strategies. If correct, herein lays a unique and rich opportunity to leverage pet dogs and cats with immunogenic cancers for the evaluation of innovative combinatorial strategies for the dual purpose of advancing veterinary patient care, but also guiding scientific discovery and clinical practice recommendations for comparative tumor types afflicting human beings.

## Author Contributions

SR drafted original article and generation of figures. TF revised article and modified figures. All authors contributed to the article and approved the submitted version.

## Funding

This review article was supported by Morris Animal Foundation award D20CA-605.

## Conflict of Interest

The authors declare that the research was conducted in the absence of any commercial or financial relationships that could be construed as a potential conflict of interest.

## Publisher’s Note

All claims expressed in this article are solely those of the authors and do not necessarily represent those of their affiliated organizations, or those of the publisher, the editors and the reviewers. Any product that may be evaluated in this article, or claim that may be made by its manufacturer, is not guaranteed or endorsed by the publisher.
